# Identifying temporal and spatial patterns of variation from multimodal data using MEFISTO

**DOI:** 10.1038/s41592-021-01343-9

**Published:** 2022-01-13

**Authors:** Britta Velten, Jana M. Braunger, Ricard Argelaguet, Damien Arnol, Jakob Wirbel, Danila Bredikhin, Georg Zeller, Oliver Stegle

**Affiliations:** 1 Division of Computational Genomics and Systems Genetics, German Cancer Research Center (DKFZ), Heidelberg, Germany; 2 Cellular Genetics Programme, Wellcome Sanger Institute, Cambridge, UK; 3 European Molecular Biology Laboratory, European Bioinformatics Institute (EMBL-EBI), Cambridge, UK; 4 Epigenetics Programme, Babraham Institute, Cambridge, UK; 5 European Molecular Biology Laboratory, Structural and Computational Biology Unit, Heidelberg, Germany; 6 European Molecular Biology Laboratory, Genome Biology Unit, Heidelberg, Germany; 7 Collaboration for joint PhD degree between EMBL and Heidelberg University, Faculty of Biosciences, Heidelberg University, Heidelberg, Germany

**Keywords:** Software, Statistical methods, Transcriptomics, Machine learning, Microbial communities

## Abstract

Factor analysis is a widely used method for dimensionality reduction in genome biology, with applications from personalized health to single-cell biology. Existing factor analysis models assume independence of the observed samples, an assumption that fails in spatio-temporal profiling studies. Here we present MEFISTO, a flexible and versatile toolbox for modeling high-dimensional data when spatial or temporal dependencies between the samples are known. MEFISTO maintains the established benefits of factor analysis for multimodal data, but enables the performance of spatio-temporally informed dimensionality reduction, interpolation, and separation of smooth from non-smooth patterns of variation. Moreover, MEFISTO can integrate multiple related datasets by simultaneously identifying and aligning the underlying patterns of variation in a data-driven manner. To illustrate MEFISTO, we apply the model to different datasets with spatial or temporal resolution, including an evolutionary atlas of organ development, a longitudinal microbiome study, a single-cell multi-omics atlas of mouse gastrulation and spatially resolved transcriptomics.

## Abstract (web summary)

MEFISTO models bulk and single-cell multi-omics data with temporal or spatial dependencies for interpretable pattern discovery and integration.

## Main

Factor analysis is a first-line approach for the analysis of high-throughput sequencing data^1–4^, and is increasingly applied in the context of multi-omics datasets^5–8^. Given the popularity and broad applicability of factor analysis, this model class has undergone an evolution, from principal component analysis to sparse generalizations^4^, including non-negativity constraints^2,3,9^. Most recently, factor analysis has been extended to model structured datasets that consist of multiple data modalities or sample groups^7,8^. At the same time, the complexity of multi-omics designs is constantly increasing and, in particular, strategies for assaying multiple omics layers across temporal or spatial trajectories are gaining relevance. However, existing factor analysis methods do not account for the spatio-temporal dependencies between samples that result from such designs. Prominent domains in which spatio-temporal profiling is used include developmental biology^10^, longitudinal profiling in personalized medicine^11^ or spatially resolved omics^12^. Such designs and datasets pose new analytical challenges and opportunities, including the need to account for spatio-temporal dependencies across samples that are no longer invariant to permutations; deal with imperfect alignment between samples from different data modalities, and missing data; identify inter-individual heterogeneities of the underlying temporal and/or spatial functional modules; and distinguish spatio-temporal variation from non-smooth patterns of variations. In addition, spatio-temporally informed dimensionality reduction could enable more accurate and interpretable recovery of the underlying patterns by leveraging known spatio-temporal dependencies rather than by solely relying on feature correlations. To this end, we propose MEFISTO, a flexible and versatile method for addressing these challenges while maintaining the benefits of previous factor analysis models for multimodal data.

## Results

MEFISTO takes as input a dataset that contains measurements from one or more feature sets (for example, different omics), referred to as “views” in the following, as well as one or multiple sets of samples (for example, from different experimental conditions, species or individuals), referred to as “groups” in the following. In addition to these high-dimensional data, each sample is further characterized by a continuous covariate such as a one-dimensional temporal or two-dimensional spatial coordinate. MEFISTO factorizes the input data into latent factors, similar to conventional factor analysis, thereby recovering a joint embedding of the samples in a low-dimensional latent space. At the same time, the model yields a sparse linear and therefore interpretable mapping between the latent factors and the observed features in terms of view-specific weights. Formulated within a probabilistic framework, MEFISTO naturally accounts for missing values for arbitrary combinations of views, groups and covariate values.

**Fig. 1:**
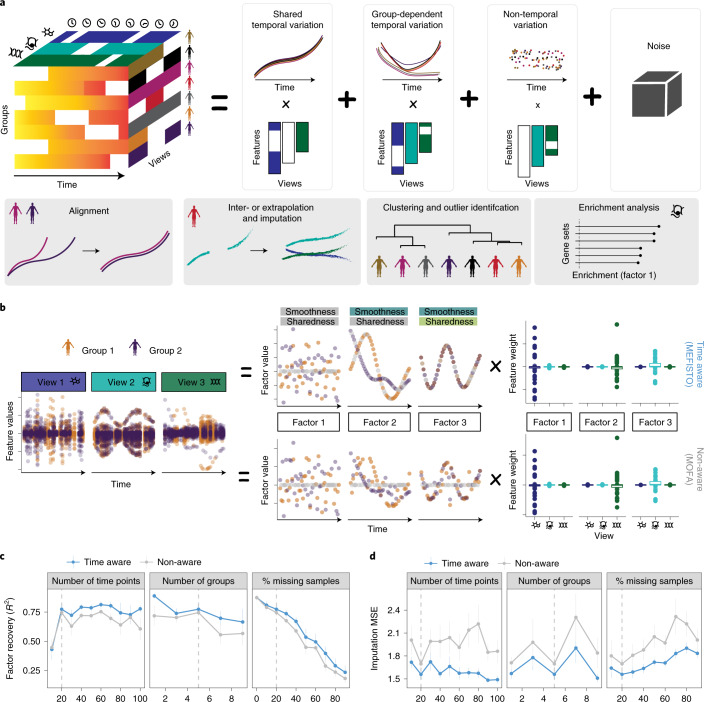
Overview of MEFISTO. **a**, Illustration of MEFISTO for time-resolved data: MEFISTO decomposes a high-dimensional dataset with measurements from multiple views (for example, omics, tissues, genomic regions), sample groups (for example, individuals, biological conditions, species) and time points into a small number of factors in a time-aware manner. The inferred factors can explain temporally smooth variation that is shared across sample groups, smooth variation that is specific to sample groups or non-smooth variation. The boxes below illustrate additional features of MEFISTO, including data-driven alignment between misaligned sample groups, interpolation and imputation of missing data, clustering and outlier identification and enrichment analysis to annotate factors. **b**, Comparison of MEFISTO with conventional factor analysis that is not aware of time (MOFA) using simulated data. Shown are results from the application of both models to a simulated dataset with one non-smooth factor (Factor 1), one smooth, non-shared factor (Factor 2) and one smooth, shared factor (Factor 3). **c**,**d**, Recovery of the latent factors (Pearson *R*^2^) (**c**) and the imputation performance on missing values (mean squared error (MSE)) (**d**) for varying number of time points, groups and levels of missingness in the comparison of MEFISTO and MOFA on simulated data. Shown are the mean and standard error of the mean estimated across 10 independent repeat experiments. The dashed vertical line denotes the base parameter value kept constant when varying other parameters (Methods).

Unlike existing factor analysis methods for multimodal data, MEFISTO incorporates the continuous covariate to account for spatio-temporal dependencies between samples, which allows for the identification of both spatio-temporally smooth factors as well as non-smooth factors that are independent of the continuous covariate (Fig. 1a,b). Technically, MEFISTO combines factor analysis with the flexible non-parametric framework of Gaussian processes^13^ to model spatio-temporal dependencies in the latent space, where each factor is governed by a continuous latent process with a variable degree of smoothness (Supplementary Information). Gaussian processes have previously been used in biomedical applications to encode temporal or spatial proximity^14–18^, however, so far they have been used primarily for univariate data (see Methods for an overview on existing use cases).

For experimental designs with repeated spatio-temporal measurements, for example, longitudinal studies that involve multiple individuals, species or experimental conditions, MEFISTO models and accounts for heterogeneity across these groups of samples, thereby inferring the extent to which spatio-temporal patterns are shared across groups (referred to as “sharedness”, Fig. 1b). To cope with imperfect alignment across groups, MEFISTO comes with an integrated data-driven alignment step of the temporal covariate by combining the inference of the latent space with dynamic time warping^19^. In brief, MEFISTO learns a non-linear monotonic warping function based on the major sources of variation across all views as captured in the latent space (Supplementary Information), and thereby provides a correspondence between time points across sample groups.

To enable efficient inference in large datasets, MEFISTO leverages sparse Gaussian process approximations^20^, as well as efficient Kronecker decompositions if a common spatio-temporal sampling is present across groups^21^ (Supplementary Information). Once fitted, the model allows for different downstream analyses (Fig. 1a), including imputation as well as interpolation and extrapolation along the spatio-temporal axis. It also allows for identification of molecular signatures that underlie the latent factors, as well as clustering and outlier identification at the level of samples (for example, the measurement at a single time point), as well as groups of samples (for example, an individual with distinct temporal trajectories).

### Validation using simulated data

**Extended Data Fig. 1:**
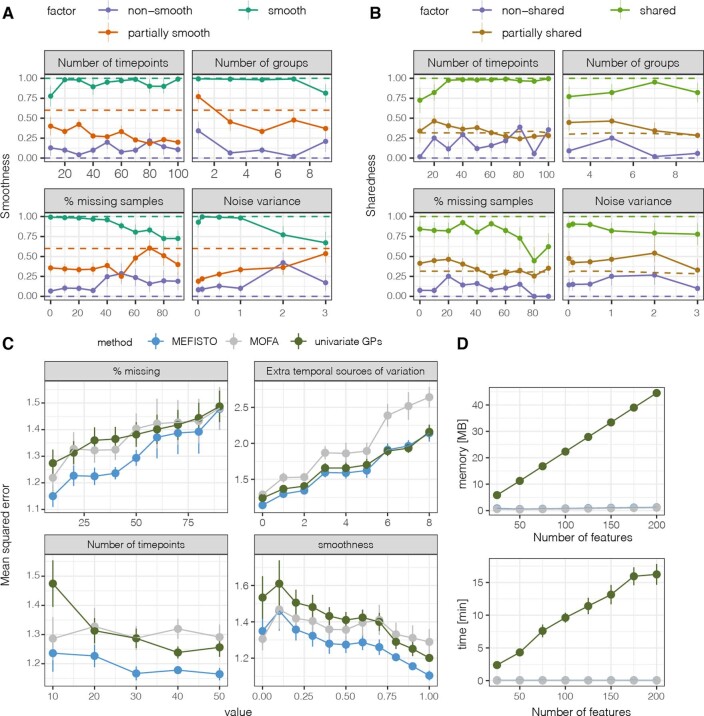
Additional results from evaluating MEFISTO on simulated data. (**a**, **b**) Assessing the inference of factor smoothness (**a**) and sharedness (**b**, as defined based on the covariance of a factor across groups, Methods) on simulated data for varying simulation parameters (panels, Methods). Solid lines and dots show the average scores inferred by MEFISTO, intervals indicate the standard error of the mean across ten independent trials and dashed lines the values used in the simulation per factor (colors). (**c**,**d**) Comparison of interpolation performance to univariate Gaussian processes in terms of mean squared error of imputation (**c**) and memory and time requirements (**d**) for varying simulation parameters (panels, Methods). Dots indicate mean, intervals indicate standard error of the mean across ten independent trials.

Initially, we considered simulated time course data drawn from the generative model of MEFISTO with multiple views and sample groups to validate the model (Methods). We assessed MEFISTO in terms of recovery of the true latent factors, imputation of missing values in the input data, as well as estimation of the smoothness and sharedness of each factor. For comparison we also considered MOFA^7,8^, a multimodal factor analysis model that does not take the temporal covariate into account. Over a range of simulated settings, MEFISTO yielded improved recovery of the latent space and offered more accurate imputation of missing data (Fig. 1c,d). Moreover, MEFISTO correctly estimated the smoothness and sharedness of individual factors, thereby enabling temporal variation to be distinguished from non-temporal variation (Extended Data Fig. 1a) and identification of the extent to which temporal patterns were shared across groups (Extended Data Fig. 1b). Additionally, MEFISTO was robust to misaligned time points across groups, correctly recovering the true sample alignment (Supplementary Figs. 1–3). We also compared the imputation and interpolation performance of MEFISTO to univariate Gaussian process regression (Methods), finding that MEFISTO is complementary to such strategies and in particular allows for the sharing of evidence across views (Extended Data Fig. 1c,d). Finally, we assessed the computational complexity of MEFISTO, finding that the sparse Gaussian process approximations used enable applications to larger datasets (Supplementary Fig. 4).

### Application to a gene expression atlas of development

**Fig. 2:**
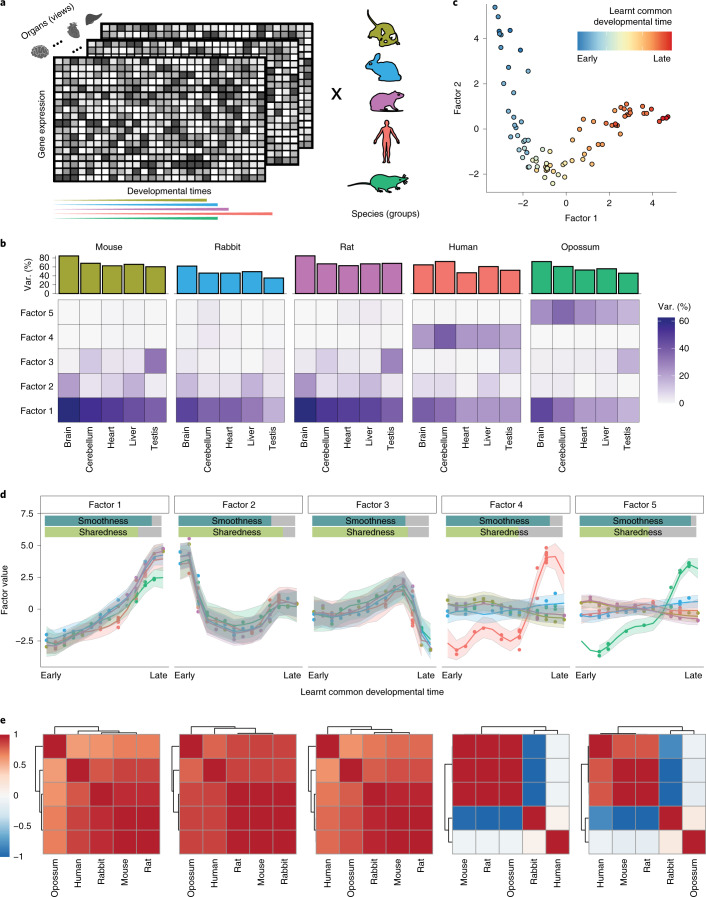
Application of MEFISTO to an evolutionary gene expression atlas across development. **a**, Illustration of the input data covering gene expression measurements for 7,696 orthologous genes from five species (groups) and five organs (views) across 14–23 developmental stages. Correspondences of stages between species are not given and are learnt by the model. **b**, Percentage of variance (var.) explained by MEFISTO in the gene expression data for each species and organ. The barplot (top) shows the percentage of variance explained by all of the factors, and the heatmap (bottom) shows the values for individual factors. **c**, Scatterplot showing the embedding of the samples given by the first two factors. Samples are colored by the inferred common developmental time. **d**, Learnt factor values as a function of the inferred developmental time. Points correspond to individual factor values, and the lines and shaded zones correspond to the mean and variance, respectively, of the underlying latent process that generates the factor values. The bars at the top indicate the estimated smoothness along development and the sharedness across species of the factor. **e**, Learnt correlation structure across species for each latent factor in **d**.

**Extended Data Fig. 2:**
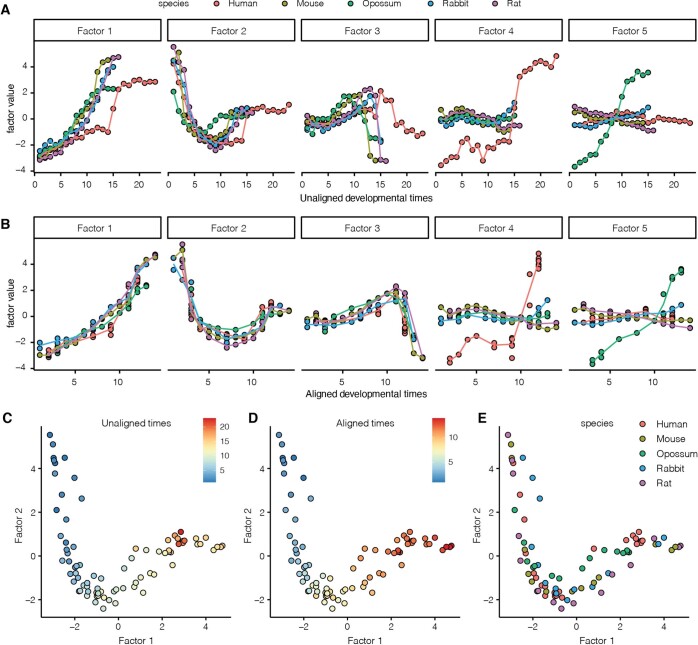
Inferred alignment of developmental stages in the evo-devo application. Factor values as a function of time before (**a**) and after (**b**) alignment. (**a**) shows the factor values (y-axis) against the developmental stages without alignment across species (x-axis), (**b**) shows the factor values (y-axis) against the developmental stages with alignment across species (x-axis). (**c**,**d**,**e**) show a latent embedding given by the factor values for each species- time point combination for Factor 1 (x-axis) and Factor 2 (y-axis) colored by unaligned times (**c**), aligned times (**d**) and species (**e**).

Next, we applied MEFISTO to an evolutionary atlas of mammalian organ development^10^ (Fig. 2a), consisting of gene expression of five species (that is, groups) profiled across five organs (that is, views) along a developmental time course from early organogenesis to adulthood (14–23 time points per species). MEFISTO identified five latent factors that were robust to down-sampling of time points (Supplementary Fig. 5) and which collectively explained 35–85% of the transcriptome variation for different organs (Fig. 2b). Despite a substantial fraction of missing time points for several combinations of organs and species (Supplementary Fig. 6), the temporal alignment of MEFISTO (Fig. 2c and Extended Data Fig. 2) yielded meaningful correspondence of the developmental stages between species (Supplementary Fig. 7). All five factors were characterized by a high degree of smoothness (Fig. 2d), which is consistent with developmental programs driving most of the variation. Notably, the sharedness across species varied considerably between factors (Fig. 2d).

**Extended Data Fig. 3:**
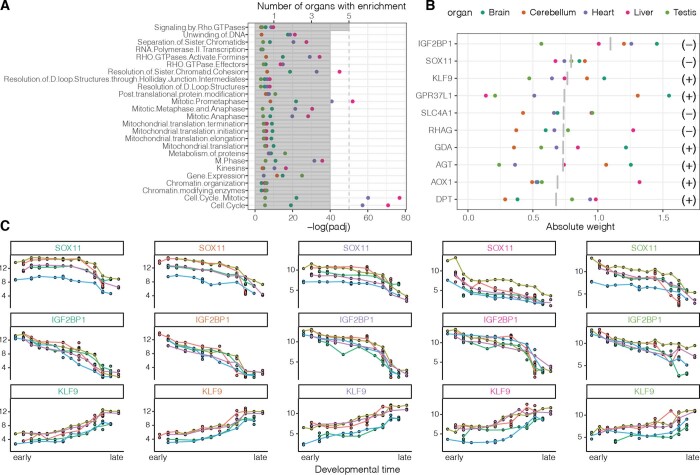
Pan-organ developmental programs on Factor 1 in the evo-devo application. (**a**) Gene sets at a false discovery rate of 5% that are enriched in the weights of Factor 1 in at least 4 organs. Dots are colored by organ and indicate the significance of a gene set (x-axis) based on a parametric t-test with multiple testing correction using Benjamini-Hochberg procedure as implemented in *MOFA2*. Gray bars indicate the number of organs with significant enrichment. (**b**) Top 10 genes (y-axis) with highest absolute mean weight across organs. Dots indicate the absolute weight per organ (colors), gray bars show the mean across organs. Symbols on the right indicate the sign of the weights. (**c**) Gene expression along the inferred developmental time in all organs (columns) for the top 3 genes of panel (**b**).

**Extended Data Fig. 4:**
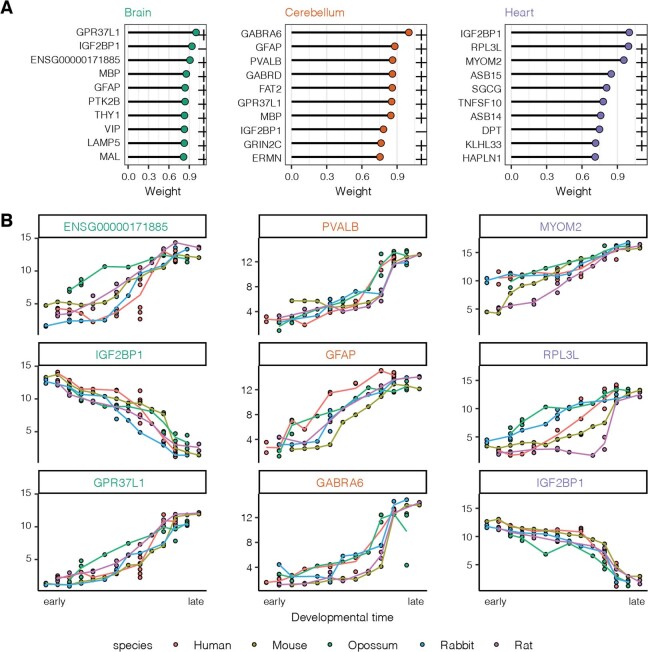
Organ-wise weights of Factor 1 in the evo-devo application. (**a**) Genes with highest absolute weight (x-axis) for the three organs with highest variance explained by Factor 1. Symbols on the right in each panel indicate the sign of the weight. (**b**) Gene expression trajectories along the inferred developmental time for the top 3 genes of the corresponding panel in (**a**).

**Extended Data Fig. 5:**
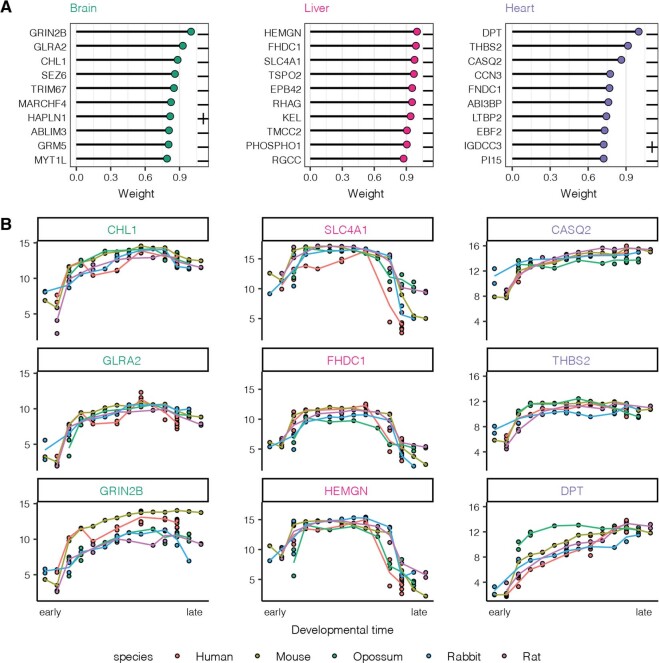
Organ-wise weights of Factor 2 in the evo-devo application. (**a**) Genes with highest absolute weight (x-axis) for the three organs with highest variance explained by Factor 2. Symbols on the right in each panel indicate the sign of the weight. (**b**) Gene expression trajectories along the inferred developmental time for the top 3 genes of the corresponding panel in (**a**).

**Extended Data Fig. 6:**
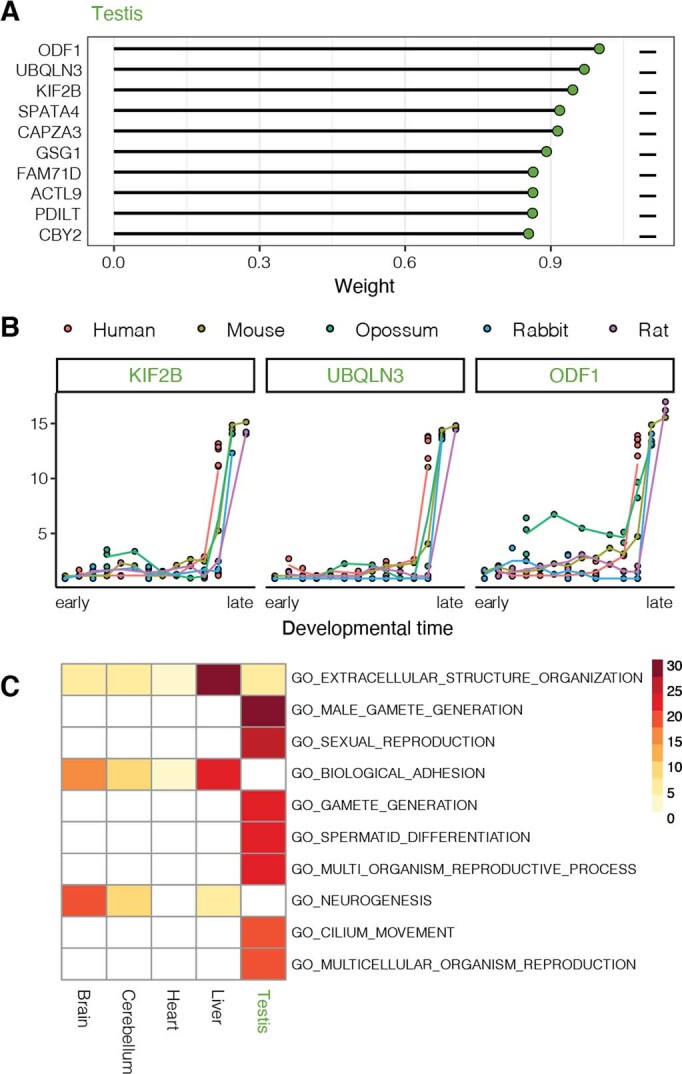
Testis weights of Factor 3 in the evo-devo application. (**a**) Genes with highest absolute weight (x-axis) in Testis on Factor 3. Symbols on the right indicate the sign of the weight. (**b**) Gene expression trajectories along the inferred developmental time for the top 3 genes in (**a**). (**c**) Top ten enriched gene set of the Molecular Signatures Database (MSigDB) in the weights of Factor 3. Colors indicate the negative logarithm of the adjusted p-values (per organ and factor) based on a parametric t-test with multiple testing correction using Benjamini-Hochberg procedure as implemented in *MOFA2*.

The first three factors had similar temporal profiles across species, indicating that they captured conserved developmental programs. Factor 1 explained variation in all organs (Fig. 2b), capturing gradual expression changes along developmental time (Fig. 2d). To further characterize the underlying molecular process, we investigated the genes with high weights on the factor. Across all organs this showed gene sets linked to broad developmental processes and proliferation, including pathways related to the cell cycle (Extended Data Fig. 3a), but also individual genes encoding hallmark developmental modulators such as IGF2BP1, SOX11 or KLF9^22–24^ (Extended Data Fig. 3b,c). At the same time, the weights of Factor 1 also indicated organ-specific signatures that varied in line with the major functions of the respective organ, for example, upregulation of *GFAP* expression along Factor 1 in brain tissues (Extended Data Fig. 4)^25^. Similarly, Factor 2 explained variation in multiple organs (Fig. 2b) and captured developmental programs with onset in intermediate development (Fig. 2d), as for example characterized by a transient upregulation of *HEMGN* expression during development in the liver along Factor 2 (Extended Data Fig. 5). Factor 3 captured gene expression signatures specific to testis development, with a sharp transition in gene expression with the onset of male meiosis (Fig. 2b,d). As visible from the factor weights, these signatures are characterised by expression changes in genes encoding testis-specific proteins, for example, ODF1 or UBQLN3, which are upregulated in testis at late developmental stages (Extended Data Fig. 6a,b), and in gene sets linked to reproduction (Extended Data Fig. 6c).

**Extended Data Fig. 7:**
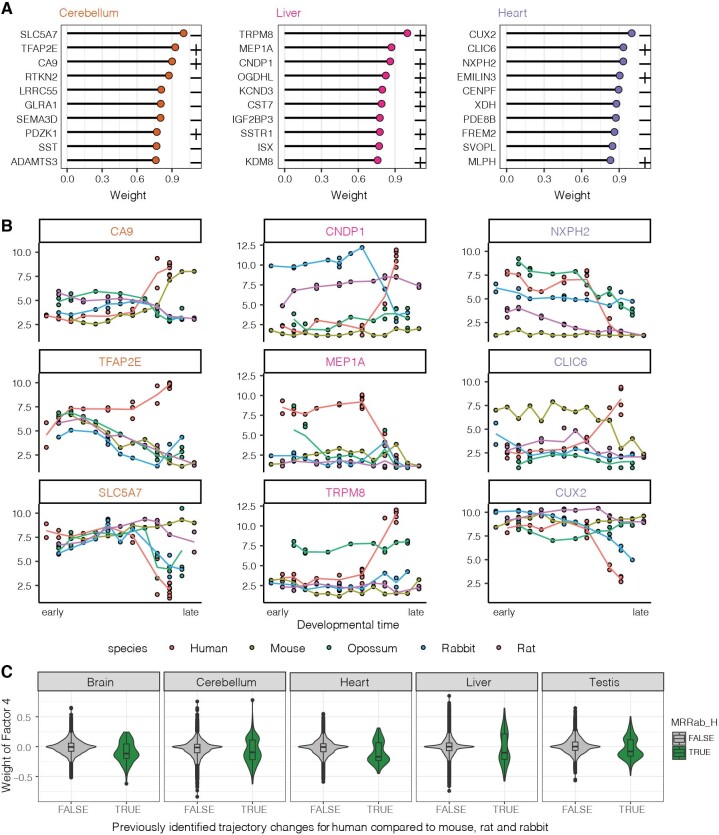
Organ-wise weights of Factor 4 in the evo-devo application. (**a**) Genes with highest absolute weight (x-axis) for the three organs with highest variance explained by Factor 4. Symbols on the right in each panel indicate the sign of the weight. (**b**) Gene expression trajectories along the inferred developmental time for the top 3 genes of the corresponding panel in (**a**). (**c**) Weights of Factor 4 split by the classification in Cardoso-Moreira et al^10^. Shown are violin plots of the weights (n = 7,696) in the model for each organ (panels) separated by whether they have previously been identified as having changed developmental trajectories for human compared to rodents or rabbit (x-axis). Inner boxplots show the median, the first and third quartiles (box), the largest and smallest value within the 1.5 interquartile ranges from the hinges (end of whiskers) and outliers (dots).

**Extended Data Fig. 8:**
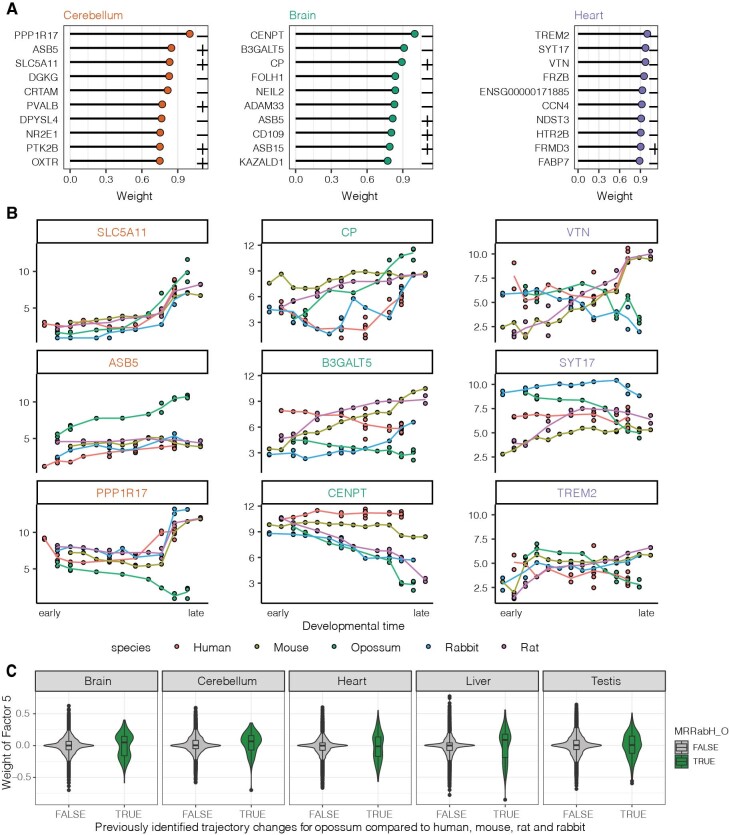
Organ-wise weights of Factor 5 in the evo-devo application. (**a**) Genes with highest absolute weight (x-axis) for the three organs with highest variance explained by Factor 5. Symbols on the right in each panel indicate the sign of the weight. (**b**) Gene expression trajectories along the inferred developmental time for the top 3 genes of the corresponding panel in (**a**). (**c**) Weights of Factor 5 split by the classification in Cardoso-Moreira et al^10^. Shown are violin plots of the weights (n = 7,696) in the model for each organ (panels) separated by whether they have previously been identified as having changed developmental trajectories for opossum compared to the other mammals (x-axis). Inner boxplots show the median, the first and third quartiles (box), the largest and smallest value within the 1.5 interquartile ranges from the hinges (end of whiskers) and outliers (dots).

In addition to these shared factors, MEFISTO identified variation specific to the evolutionarily more distant species human (Factor 4) and opossum (Factor 5), with distinct temporal patterns (Fig. 2d,e). Interestingly, these two factors affect gene expression programs in all organs (Fig. 2b and Extended Data Figs. 7,8). To identify individual genes that have undergone changes to the expression trajectory along evolution, we inspected the factor weights for each organ. Several of the genes with high weights were previously associated with differences in expression trajectory that have evolved on branches separating opossum and human from the other species^10^ (Extended Data Fig. 7c and Extended Data Fig. 8c). Most of these genes had a high factor weight only in one of the organs (Supplementary Fig. 8a,b), which is in line with previous findings that the majority of trajectory changes are restricted to one organ^10^. These changes are probably caused by regulatory mutations or changes in cell type composition that occurred in this organ^10^. For example, evolutionary changes in primates have been reported for *TRPM8*^26^, which was assigned the highest weight in the liver on the human-specific Factor 4 (Extended Data Fig. 7a,b). Moreover, neutrophil markers^27^ were enriched in genes with high weights for the opossum-specific Factor 5 (Supplementary Fig. 8c), indicating cell type composition changes in line with previously observed differences in the developmental timing of neutrophils in marsupials^28^.

Finally, we considered this dataset to further assess the performance of MEFISTO in settings with pronounced missingness by masking data for random species–time point combinations. MEFISTO yielded accurate imputations, and in particular was able to interpolate time points with completely missing data (Supplementary Fig. 9), while leveraging both temporal information and correlations between features for imputation (Supplementary Fig. 10).

### Application to sparse longitudinal microbiome data

**Fig. 3:**
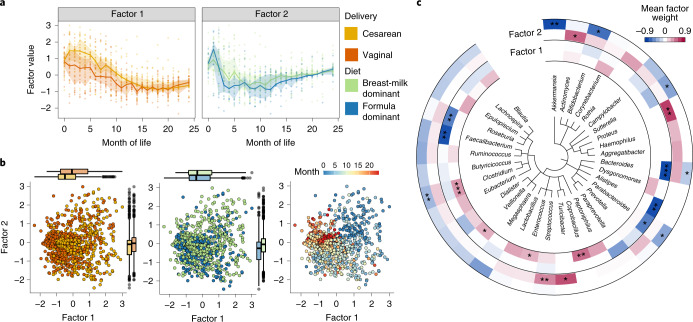
Application to a longitudinal microbiome study following infants after birth. **a**, Factor values as a function of month of life colored by delivery mode (left, Factor 1) and predominant feeding mode, termed diet (right, Factor 2). Dots represent inferred factor values per infant; lines correspond to the median across all samples in the respective category with the shaded zones indicating the interquartile range. **b**, Scatterplot of Factor 1 versus Factor 2 across samples, with colors denoting delivery mode (left), diet (middle) and month of life (right). Boxplots show the median (black horizontal line), the first and third quartiles (ends of the box), the largest and smallest value within the 1.5 interquartile ranges (ends of the whiskers) and the outliers (dots) for the *n* = 1,032 factor values of the 43 infants (groups) and 24 time points. **c**, Taxonomic tree annotated by mean positive and negative weights for Factor 1 and 2. Shown are genera with at least three sOTUs. Significance of enrichment is given as *adjusted *P* < 0.05, **adjusted *P* < 0.01 and ***adjusted *P* < 0.001 (one-sided Wilcoxon test, adjusted for multiple testing, Methods).

As a second use case, we applied MEFISTO to longitudinal samples of the microbiome of infants after birth^29,30^ using month of life as the temporal covariate and infants as the groups in the model. As common in microbiome data and longitudinal studies, this dataset is extremely sparse, with 91.4–98.0% of the dataset consisting of zeros and up to 23 missing time points per infant (out of 24 time points; 9 time points missing on average). MEFISTO identified distinct temporal trajectories depending on the birth mode (Factor 1, Fig. 3a) and, to a lesser extent, the diet of the infants (Factor 2, Fig. 3a). Unlike methods that do not account for the temporal covariate, MEFISTO yielded robust estimates of factor values when masking randomly selected subsets of the samples (Supplementary Fig. 11). Taken together, these two factors explained between 6% and 61% of the total microbiome variation in each infant, and had a clustering that primarily captured temporal effects at the level of samples (Fig. 3b) and delivery mode at the level of infants (Factor 1, Supplementary Fig. 12). To identify specific changes in the microbiome that underlie the temporal patterns captured by the factors, we investigated the weights of the microbial features (that is, sub-operational taxonomic units (sOTUs)) in the model. For Factor 1, the genera with the largest weights were *Faecalibacterium* and *Bacteroides*, which were negatively associated with factor activity (Fig. 3c). In line with the temporal pattern of Factor 1 (Fig. 3a), these genera play an important role in the maturation of the human gut microbiome and become increasingly abundant over the course of the first year of life, reaching stable abundance levels in the second year^29,31^. Moreover, the higher values of Factor 1 over the first year of life indicate that microbiome maturation is slower in infants born by cesarean section (Fig. 3a), in whom colonization towards an adult microbiome is known to be delayed compared with vaginally delivered infants^29,31^. For example, *Bacteroides*, as captured by negative factor weights (Fig. 3c), is more abundant in vaginally delivered infants in the early months after birth^29,31^. In contrast, *Clostridium*, enriched in positive factor weights (Fig. 3c), is predominantly observed in infants delivered by cesarean section (Supplementary Fig. 13a,b) and decreases in abundance over the course of the first 1.5 years during the development of a mature gut microbiome^29,31^. sOTUs with high weights on Factor 2 were associated with the diet of infants (Supplementary Fig. 13a,c), including an enrichment of Clostridiales for the formula diet, which might reflect a more adult-like diet and lack of oligosaccharides from human breast milk. At the same time Factor 2 captured microbes with sharp changes in abundance in the first months after delivery, such as the decline in abundance of *Proteus* on the positive weights (Fig. 3c) and an increase in abundance of *Bifidobacterium* on the negative weights (Fig. 3c). We also compared MEFISTO with a recently proposed method for temporal analysis of microbiome data (CTF)^30^, which yielded factors that were notably less concordant with the expected axes of microbiome variation in these data (Supplementary Fig. 13a), and had no clear taxonomic enrichment in the factor weights (Supplementary Fig. 13d).

### Applications to multi-dimensional and spatial omics

**Fig. 4:**
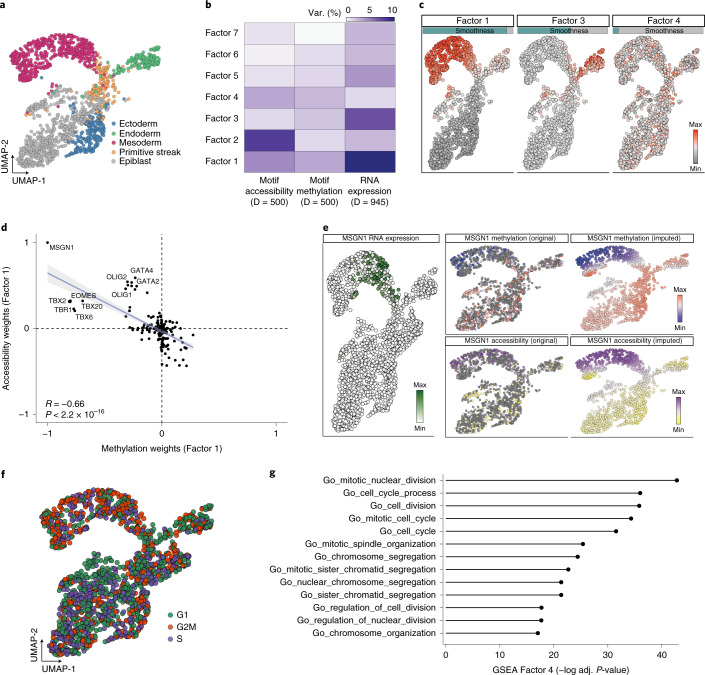
Application to a single-cell multi-omics dataset from early mouse development. **a**, Scatterplots of UMAP (uniform manifold approximation and projection for dimension reduction) coordinates obtained from the RNA expression data that were used as covariates for MEFISTO. Each dot corresponds to a cell, colored by lineage assignments derived from the Argelaguet el al. study^32^. **b**, Percentage of variance explained by each factor in each data modality. **c**, Scatterplot of UMAP coordinates as in **a**, colored by factor values. The bars at the top indicate the estimated smoothness of the respective factor. **d**, Scatterplot of DNA methylation weights versus chromatin accessibility weights for Factor 1 (relative values). Each dot corresponds to a transcription factor motif, error bands indicate the 95% confidence interval of the linear regression. Highlighted are the transcription factor motifs with the largest absolute values. Shown in the corner is Pearson *R*. The *P* value is based on a two-sided correlation test on the Pearson’s product moment correlation coefficient. **e**, Molecular variation of MSGN1 along the trajectory. Left: RNA expression level. Middle: DNA methylation (top) and chromatin accessibility (bottom) raw data values (~33% of cells covered). Right: DNA methylation (top) and chromatin accessibility estimates (bottom) using imputed values obtained from MEFISTO. **f**, Scatterplots of UMAP coordinates, as in **a**. Each cell is colored by cell cycle state, inferred using *cyclone*^37^. **g**, Gene set enrichment analysis (GSEA) applied to the RNA weights of Factor 4. Shown is the false discovery rate-adjusted *P* value for the top significant pathways from the Molecular Signatures Database^38^.

Finally, we considered MEFISTO for the analysis of datasets with a multi-dimensional covariate. We applied MEFISTO to a single-cell multi-omics study^32^ consisting of 1,518 cells collected across early mouse development that were profiled using combined nucleosome, methylation and transcriptome sequencing (scNMT-seq^33^) or transcriptome sequencing. The sparsity and missing data of the epigenetic readouts is a major challenge in this dataset, with only 33% of the cells having measurements from the epigenetic modalities. To identify coordinated variation between the transcriptome and epigenome along development, we characterized developmental transitions using two-dimensional reference coordinates derived from the RNA expression (Fig. 4a, UMAP^34^) and used these as covariates in MEFISTO (Methods). Applied to all three omics layers, and considering DNA methylation and chromatin accessibility quantified at transcription factor motifs as input (Methods), MEFISTO identified seven factors that jointly explained 29%, 35% and 39% of the variance in RNA expression, DNA methylation and chromatin accessibility, respectively (Fig. 4b). Factors 1 and 3 captured smooth patterns of variation across all data modalities, associated with the emergence of the two primary germ layers, mesoderm (Factor 1) and endoderm (Factor 3) (Fig. 4c). The weights of the transcription factor motifs on these factors reflected the known negative relationship of DNA methylation and chromatin accessibility^35^ and identified key transcription factors associated with this process, including GATA4, TBX6 and MSGN1 for the mesoderm fate (Fig. 4d) and FOXA2 and HNF1 for the endoderm fate (Supplementary Fig. 14a). Notably, MEFISTO inferred additional non-smooth factors that captured biological sources of covariation not associated with the developmental trajectory. The most prominent example is Factor 4, which captured differences in cell cycle state (Fig. 4c,f, Methods), with an enrichment of weights in the RNA view for gene sets related to the cell cycle (Fig. 4g). Finally, we used the underlying Gaussian processes inferred by MEFISTO to denoise transcription factor activities and impute accessibility and methylation values of transcription factor motifs in cells for which only RNA expression measurements were available (Fig. 4e and Supplementary Figs. 14b,15). This analysis illustrates the ability of MEFISTO to impute entire molecular layers along multi-dimensional trajectories, which is particularly valuable for the analysis of very sparse data types such as single-cell multi-omics technologies. In conclusion, this application shows how MEFISTO can be applied to noisy and complex single-cell multi-omics datasets to identify coordinated transcriptomic and epigenetic signatures in multi-dimensional trajectories.

**Extended Data Fig. 9:**
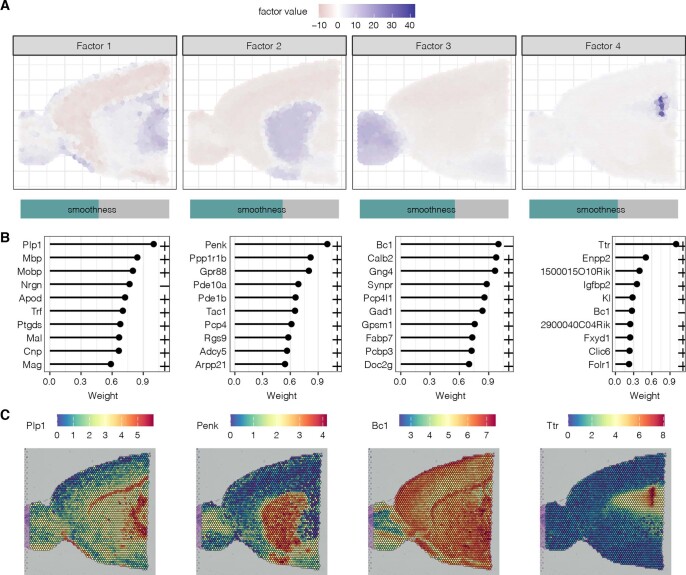
Application to spatial transcriptomics data. (**a**) Recovered factor values across space. The x- and y-axis denote the spatial coordinates, the colors indicate the inferred factor values. Bars below show the inferred smoothness scores for each factor. (**b**) Genes with highest absolute weight for the corresponding factor in (**a**). Symbols on the right of each panel indicate the sign of the weight. (**c**) Normalized gene expression values (colors) across space for the gene with the highest absolute weight on the corresponding factor in (**a**).

Similarly, MEFISTO can be used to identify spatial patterns. To illustrate this, we applied MEFISTO to a 10x Visium spatial transcriptomics dataset of the anterior part of the mouse brain^36^ using the spatial coordinates as the covariate in the model. MEFISTO identified major anatomical regions in the brain (Extended Data Fig. 9a) and their associated marker genes (Extended Data Fig. 9b,c), such as *Ttr* as a marker of the choroid plexus (Factor 4), without the need of single-cell reference data. Enrichment analysis of the weights based on known marker genes (Methods) showed cell types enriched for each of the patterns, including Schwann cells on Factor 1, neuroendocrine cells on Factor 2, Purkinje neurons on Factor 3 and choroid plexus cells on Factor 4 (Supplementary Fig. 16). MEFISTO provides an integrated measure of the smoothness of each pattern across space (Extended Data Fig. 9a). This application also illustrates the utility of the sparse inference scheme in MEFISTO, which greatly reduces time and memory requirements while retaining accurate inference of the spatial patterns as well as interpolation to missing spots (Supplementary Fig. 17).

## Discussion

Here, we present MEFISTO, a computational framework that opens up the application of multimodal factor analysis to temporal or spatially resolved datasets. We found that the ability to explicitly account for spatial or temporal dependencies is especially helpful in datasets with a larger number of missing values, or when high-dimensional measurements are sampled irregularly across different sample groups or views. Additionally, MEFISTO adds substantial value in cases in which extra- or interpolation of temporal or spatial trajectories is required and/or when the temporal covariate and the associated measures are imperfectly aligned across datasets. We focused on applications of MEFISTO to temporal and longitudinal studies, such as developmental time courses. These studies are rapidly gaining relevance both in basic biology and biomedicine. However, the model is also readily applicable to two-dimensional covariates, as illustrated in the application to multimodal single-cell data and the application to Visium gene expression arrays.

Future developments could focus on extensions to enable spatial alignment across datasets, as well as the deployment of specific noise models. These could, for example, be tailored for single-molecule data, directly account for over-dispersion in sequencing data without the need for preprocessing, or help to distinguish biological and technical zeros in the measurements by incorporating an explicit model of zero-inflation. Furthermore, although MEFISTO is based on a probabilistic factor analysis framework, the concept of explicitly modeling spatial and temporal covariates could also be incorporated into other classes of latent variable models. This includes, for example, non-negative matrix factorization, which has been successfully applied to recover additive non-negative signatures, or autoencoders, which are increasingly used to infer a non-linear decomposition of the data. Finally, we note that beyond time or space, other side-information could be considered to inform the factorization, including clinical markers or known dependencies between molecular features.

## Methods

### MEFISTO model

MEFISTO is a probabilistic model for factor analysis that accounts for continuous side-information during inference of the latent space. To achieve this, MEFISTO combines multimodal sparse factor analysis frameworks^7,8^ with a functional view on the latent factors based on Gaussian processes, and additionally provides alignment functionalities and an explicit model of intergroup heterogeneity. As input MEFISTO expects a collection of matrices, where each matrix ${\bf{Y}}^{m,g}$ corresponds to a group *g* =1,…,*G* and view *m* =1,…,*M* with *N*_*g*_ samples in rows and *D*_*m*_ features in columns. Each sample is further characterized by a covariate $\bf{C}^g \in {\Bbb R}^{C \times N_g}$ that represents, for example, temporal or spatial coordinates. The matrices are jointly decomposed as

$$\bf{Y}^{m,g} = \bf{Z}^g\bf{W}^{mT} + \bf{\it{\upvarepsilon }}^{m,g}\;\;\;\;\;m = 1, \ldots ,M,g = 1, \ldots ,G,$$

where $\bf{Z}^g \in {\Bbb R}^{N_g \times K}$ contains the *K* latent factors and $\bf{W}^m \in {\Bbb R}^{D_m \times K}$ contains their weights. A feature- and view-wise sparsity prior is used for ${\bf{W}}^m$ as in previous multimodal factor analysis models^7,8^. Unlike existing factor models, however, the model additionally accounts for the covariate ${\bf{C}}^g$. Each factor value $z_{nk}^g$ is modeled as a realization of a Gaussian process

$$z_{nk}^g = f_k\left( {\bf{c}_n^g} \right) + \eta _{nk}^g\;\;\;\;\;{{{\mathrm{with}}}}\;\,f_k\sim \mathrm{GP}\left( {0,\kappa _k} \right),$$

where the covariance function *κ*_*k*_ models the relationship between groups as well as along the covariate, that is,

$$\kappa _k\left( {\bf{c}_n^g,\bf{c}_l^h} \right) = \kappa _k^G\left( {g,h} \right)\kappa _k^C\left( {\bf{c}_n,\bf{c}_l} \right).$$

The first term in this covariance function captures the covariance of the discrete sample groups *g*, *h*, while the second term describes the covariance along values of the covariate, which provide a continuous characterization of each sample, for example, its temporal or spatial location. We choose a low-rank covariance function for 𝜅^G^ and a squared exponential covariance function for 𝜅^C^, that is,

$${\bf{K}}_k^G = \left( {\kappa _k^G\left( {g,h} \right)} \right)_{g,h} = {\bf{x}}_k {\bf{x}}_k^T + \sigma _k^2 \bf{I}\;\;\;\;\;{\bf{x}}_k \in {\Bbb R}^{G \times R}$$

$$\kappa _k^C\left( {{\bf{c}}_n,{\bf{c}}_l} \right) = s_k\exp \left( { - \frac{{||{\bf{c}}_n - {\bf{c}}_{l}||_{2}^2}}{{2l_k^2}}} \right)$$

$$\eta _{nk}^g\sim {{{\mathcal{N}}}}\left( {0,1 - s_k} \right).$$

The hyperparameters $\bf{x}_k,\sigma _k\,l_k,s_k$ determine the group–group covariance structure ($\bf{x}_k,\sigma _k$) as well as the smoothness of the latent factors along the covariate (*l*_*k*_, *s*_*k*_). The scale parameter *s*_*k*_ determines the relative smooth versus non-smooth variation per factor, and the lengthscale parameter *l*_*k*_ determines the distance over which correlation decays along the covariate, for example, in time or space. Details on the model specification, illustrations of the resulting covariance structures and a plate diagram are provided in Supplementary Information Section 2.

### Inference

To infer the unobserved model components as well as the hyperparameters of the Gaussian process, MEFISTO makes use of variational inference combined with optimization of the evidence lower bound in terms of the hyperparameters of the Gaussian processes. Details on the inference are described in Supplementary Information Section 3, where the specific updates of the inference algorithm are described. For large sample sizes, inference of the covariate kernel can be based on a subset of the original covariates chosen on a regular grid to reduce computational complexity (Supplementary Information Section 4). In addition, if the covariance matrix of the latent processes can be decomposed in terms of a Kronecker product, that is, as ${\bf{K}}^G \otimes {\bf{K}}^C$, MEFISTO leverages this structure for accelerated inference based on spectral decomposition of the group and covariate covariance (Supplementary Information Section 3).

### Alignment

If the temporal correspondence between different groups is imperfect, a non-linear alignment between sample groups is learnt based on dynamic time warping^19^ in the latent space. To reduce noise prior to the alignment, MEFISTO simultaneously decomposes the input data and aligns the covariate. This is implemented by interleaving the updates of the model components with an optimization step, in which a warping curve is found that minimizes the distance of each group to a reference group in the current latent space. The alignment can be partial, that is, it can have different end or start points between groups. Furthermore, instead of learning an alignment between individual groups, the alignment step can also be used at higher levels, such as between distinct classes of groups based on known class annotations or hierarchies of the groups. Details on the alignment step are described in Supplementary Information Section 5 and we provide practical guidelines on the use of the alignment option in Supplementary Information Section 8.3.

### Data preprocessing and model set-up

For each view a different likelihood model can be used in the matrix decomposition analogously to previous multimodal factor models (Supplementary Information Section 8.1). Nevertheless, for most data types, preprocessing of the data prior to MEFISTO is recommended to take characteristics of the data into account such as over-dispersion or differences in library size in sequencing count data. We provide a detailed discussion and guidelines in Supplementary Information Section 8.1. In addition, MEFISTO can be used with tailored choices of the groups and views in the model (Supplementary Information Section 8.2).

### Downstream analyses

Once the model is trained, the Gaussian process framework enables interpolation or extrapolation of the latent factors to unseen samples, groups or views as well as providing measures of uncertainty. Given a set of new covariate values ${\bf{c}}^*$, MEFISTO can make predictions of the corresponding latent factor values ${\bf{z}}^*$ based on the predictive distribution $p({\bf{z}}^*| {\bf{Y}})$ (Supplementary Information Section 6). Missing values of the considered features are then imputed from the model equation as in previous models^7,8^. Furthermore, the hyperparameters of the model give insights into the smoothness of a factor (*s*_*k*_, between 0 (non-smooth) and 1 (smooth)) and the group relationships specific to a latent factor (${\bf{K}}^G$) that can be used to cluster the groups or identify outliers. An overall sharedness score per factor is calculated by the mean absolute distance to the identity covariance matrix in the off-diagonal elements.

### Related methods

MEFISTO is related to previous matrix factorization and tensor decomposition methods, which, however, mostly ignore temporal information^1–8^, use it only for preprocessing^39^, or interpret it post-hoc^30^. Those models that incorporate such information do not allow multiple views (for example, omics)^40–42^ or are restricted to the same features in each view^43^. In addition, sparsity constraints, which enhance interpretability and identifiability, are not used in these models. Besides linear methods, non-linear approaches have made use of continuous side-information, for example, in the context of variational autoencoders^44,45^ or recurrent neural networks^46^. In particular, all of the above methods are incapable of handling non-aligned time courses across datasets (apart from the Duncker and Sahani method^43^) and cannot capture heterogeneity across sample groups in the latent factors. For a detailed overview on related methods we refer to Supplementary Information Section 7.1. More generally, Gaussian process models have been widely applied to account for sample dependencies at the feature level. Prior applications to biomedical data include univariate regression models for spatial expression data^14–16,47^ or time course experiments^17,48^, as well as models aimed at clustering of time series ^18,49,50^. These differ in their objective to that of MEFISTO, which uses Gaussian processes at the level of inferred factors in the latent space. For a more detailed discussion see Supplementary Information Section 7.2.

### Simulations

Data were simulated from the generative model by varying the number of time points per group in a [0,1] interval, the noise levels, the number of groups and the fraction of missing values. Ten independent datasets were simulated for each setting from the generative model with three latent processes, having scale parameters of 1, 0.6, 0 and lengthscales of 0.2, 0.1, 0. For the first two (smooth and partially smooth) factors, one was randomly selected to be shared across all groups, while for the other factor a correlation matrix between groups of rank 1 was simulated randomly based on a uniformly distributed vector. MEFISTO was compared with MOFA^7,8^ in terms of factor recovery, given by the correlation of the inferred and simulated factor values, as well as in terms of the mean squared error between imputed and ground-truth values for the masked values in the high-dimensional input data. The base settings for all non-varied parameters are 20 time points per group, five groups, four views with 500 features each, and a noise variance of 1. A total of 20% of randomly selected time points were masked per group and view, of which 50% were missing in all views. To assess the alignment capabilities of the model, data were simulated with the same set-up for three groups and the covariates were transformed before training by a linear mapping (h(t) = 0.4t + 0.3), a non-linear mapping (h(t) = exp(t)), and the identity in each group, respectively. These transformed covariates were passed to the model and the learnt alignment was compared with the ground-truth warping functions. To test the alignment in the presence of non-temporal patterns of variation, we restricted the simulation to a single smooth factor and either varied the number of non-smooth factors or restricted the smooth factor to a single view with 100 features, and varied the number of features in a second view generated by a non-smooth factor. To assess the scalability in the number of time points using sparse Gaussian processes, data were simulated from one group and with the same base parameters as above. For the comparison with univariate Gaussian processes, we fitted Gaussian process models to all observed time points of each individual feature using the ExactGP model as implemented in GPyTorch v1.4.0 (ref. ^51^) with a squared exponential covariance function, and the parameters were optimized using Adam optimizer. Feature values at missing time points were predicted from the resulting posterior. Data were simulated as above with only the two smooth factors (given that univariate Gaussian processes are restricted to modeling temporal patterns in the data), as well as a single group and 100 features per view.

### Evo-devo data

Count data were obtained from Cardoso-Moreira et al.^10^, corrected for library size, normalized using a variance stabilizing transformation provided by DESeq2 v1.26.0 (ref. ^52^) and the orthologous genes selected as given in the Cardoso-Moreira et al. study^10^. Following the trajectory analysis of the original publication, we focused on five species, namely human, opossum, mouse, rat and rabbit, and five organs, namely brain, cerebellum, heart, liver and testis. In total this resulted in a dataset of five groups (species) and five views (organs) with 7,696 features each. The number of time points for each species varied between 14 and 23. Given that developmental correspondences were unclear, we used a numeric ordering within each species ranging from 1 to the maximal number of time points in this species as input for MEFISTO and let the model infer the correspondences of time points between species. Stability analysis of the latent factors was performed by re-training the model on a down-sampled dataset, in which random selections of 1–5 time points were repeatedly masked in each organ–species combination. Gene set enrichment analysis was performed based on the reactome gene sets^53^, the Molecular Signatures Database^38^ and cell type markers downloaded from https://panglaodb.se/markers.html (ref. ^27^). To assess the imputation performance, gene expression data in 2–20 randomly selected species–time combinations (out of a total of 82) were masked in three, four or all organs and the model was retrained on these data as described above. The experiment was repeated ten times and the mean squared error was calculated on all masked values. For the comparison with univariate Gaussian processes we restricted the experiment to 1,000 randomly selected genes of mouse brain and masked a varying fraction of these features at randomly sampled time points (out of 14).

### Microbiome

Data were obtained from the Code Ocean capsule: 10.24433/CO.5938114.v1, which contains the data used in the Bokulich et al. study^29^. The processed data contained microbial features provided at the level of sub-operational taxonomic units (sOTUs) and a phylogenetic tree as detailed in the Martino et al. study^30^. All samples from infants of type Stool_Stabilizer in months 0–24 of life were included, and maternal samples were excluded. Data were processed using a robust-centered log ratio following Martino et al.^30^, which treats zero values as missing, and features that were observed in less than five samples were excluded. This resulted in a total of 43 infants (groups) with up to 24 time points (months) and 969 features that were provided as input to MEFISTO using month of life as the covariate. To calculate taxonomic enrichments of the factor weights, we used a one-sided Wilcoxon test, separately comparing positive and negative weights for each genus against the appropriate background (all positive or negative weights, respectively). Mean factor weights per genus were visualized on a taxonomic tree using iTOL v6 (ref. ^54^). For the stability analysis, we randomly masked a varying number of samples (out of 650 observed samples) and trained MOFA^7,8^, MEFISTO and CTF (gemelli v0.0.5)^30^ on the masked data. For each method, factor stability was evaluated using the Pearson correlation of the factors on the masked data to the corresponding factor on the full data. To compare the factor weights of MEFISTO to associations with known covariates we trained a linear mixed-effect (LME) model for each sOTU with time point and the covariate of interest as fixed effects and infant as the random effect. We subsequently extracted the LME model coefficient as effect size estimates and compared them to the factor weights of MEFISTO.

### Single-cell multi-omics of mouse development

Data were obtained from the Argelaguet et al.^32^ study, in which details on quality control and data preprocessing can be found. In brief, gene expression counts were quantified over protein-coding genes using the Ensembl gene annotation 87 (ref. ^55^). The read counts were log-transformed, size-factor adjusted, the top ~1,000 most variable genes selected and the number of expressed genes per cell regressed out prior to fitting the model. The UMAP algorithm^34^ was applied to the RNA expression data to infer the two-dimensional developmental coordinates used as covariates in MEFISTO. DNA methylation and chromatin accessibility data were quantified over transcription factor motifs across the genome. A position-specific weight matrix was extracted for each motif using the JASPAR database^56^ and motif occurrences in the genome were found using the Bioconductor package motifmatchr v1.12 with default options. For each cell and transcription factor motif CpG methylation and GpC accessibility counts were aggregated across all motif instances. A CpG methylation or GpC accessibility rate for each transcription factor motif and cell was calculated by maximum likelihood under a binomial model and subsequently transformed to M-values. As input to MEFISTO we selected the top 500 most variable transcription factor motifs for each data modality. Cell cycle states for each cell were inferred using cyclone^37^ (as implemented in scran v1.18). To evaluate the imputation accuracy, random sets of cells of varying size (*N* = 100, 150, 200, 250) were selected and their epigenetic data were masked. Methods were trained on the masked data and evaluated in terms of their imputation performance using the mean absolute error to the masked measurements.

### Spatial transcriptomics

Data were obtained from the SeuratData R package as stxBrain.anterior1, normalized, and the 2,000 most variable features selected using the NormalizeData and FindVariableFeatures functions provided by Seurat^36^. Normalized expression values at all 2,696 spots were provided to MEFISTO with tissue coordinates as the two-dimensional covariate. For training of MEFISTO, 1,000 inducing points were selected on a regular grid in space. For comparison a model with 500 inducing points and a model with all spots were trained and compared in terms of their inferred factors as well as in terms of their interpolation accuracy. For the latter, 250 randomly selected spots were masked in ten independent experiments and the mean squared error between predicted and true expression values of these spots was calculated for MEFISTO (trained with different numbers of inducing points) as well as for MOFA^7,8^. Cell type markers were downloaded from https://panglaodb.se/markers.html (ref. ^27^), and markers annotated for mouse brain were used for the enrichment analysis.

### Reporting Summary

Further information on research design is available in the Nature Research Reporting Summary linked to this article.

## Online content

Any methods, additional references, Nature Research reporting summaries, source data, extended data, supplementary information, acknowledgements, peer review information; details of author contributions and competing interests; and statements of data and code availability are available at 10.1038/s41592-021-01343-9.

## Supplementary information

Supplementary Information Supplementary Methods, Supplementary Figs. 1–17

Reporting Summary

## Data Availability

The evo-devo data were obtained from Cardoso-Moreira et al.^10^ and can be accessed from ArrayExpress with codes E-MTAB-6782 (rabbit), E-MTAB-6798 (mouse), E-MTAB-6811 (rat), E-MTAB-6814 (human) and E-MTAB-6833 (opossum) (https://www.ebi.ac.uk/arrayexpress/). The microbiome data are based on Bokulich et al.^29^ and can be found on Qiita (http://qiita.microbio.me), and the processed data were obtained from the ‘Code Ocean’ capsule: 10.24433/CO.5938114.v1 provided by Martino et al.^30^. The scNMT-seq data were obtained from Argelaguet et al.^32^ and the spatial transcriptomics dataset from the SeuratData package under the name “stxBrain.anterior1”. Processed data and trained models for all applications are available at 10.6084/m9.figshare.13233860.v1 as used in the tutorials at https://biofam.github.io/MOFA2/MEFISTO. Enrichment analyses were based on gene and marker sets available from the Bioconductor package MOFAdata v1.6.0 (including the Molecular Signatures Database^38^ and Reactome^53^ gene sets) and from PanglaoDB (https://panglaodb.se/); transcription factor motifs were extracted from the JASPAR database^56^.

## Extended data

are available for this paper at 10.1038/s41592-021-01343-9.

## Code availability

MEFISTO is implemented as part of the MOFA framework^7,8^, which is available as Bioconductor package MOFA2 (version 1.3.3)^57^ and at https://github.com/bioFAM/MOFA2. Installation instructions and tutorials can be found at https://biofam.github.io/MOFA2/MEFISTO. MEFISTO can also be accessed via the Python framework muon^58^. Code to reproduce all figures is available at https://github.com/bioFAM/MEFISTO_analyses. In addition, we provide vignettes on the main applications as part of the MEFISTO tutorials on https://biofam.github.io/MOFA2/MEFISTO.
