# Supplementary material for: Identifying temporal and spatial patterns of variation from multimodal data using MEFISTO
Source: Nat Methods. 2022 Jan 13;19(2):179–86. doi: 10.1038/s41592-021-01343-9 (PMC8828471; doi:10.1038/s41592-021-01343-9)
Supplement: Supplementary file 2 — Reporting Summary [file 41592_2021_1343_MOESM2_ESM.pdf]

nature research

Corresponding author(s): Britta Velten, Oliver Stegle

Last updated by author(s): Oct 6, 2021

Reporting Summary

Nature Research wishes to improve the reproducibility of the work that we publish. This form provides structure for consistency and transparency in reporting. For further information on Nature Research policies, see our Editorial Policies and the Editorial Policy Checklist.

Statistics

For all statistical analyses, confirm that the following items are present in the figure legend, table legend, main text, or Methods section.

n/a	Confirmed	
☐	☒	The exact sample size (n) for each experimental group/condition, given as a discrete number and unit of measurement
☐	☒	A statement on whether measurements were taken from distinct samples or whether the same sample was measured repeatedly
☐	☒	The statistical test(s) used AND whether they are one- or two-sided<br>*Only common tests should be described solely by name; describe more complex techniques in the Methods section.*
☐	☒	A description of all covariates tested
☐	☒	A description of any assumptions or corrections, such as tests of normality and adjustment for multiple comparisons
☐	☒	A full description of the statistical parameters including central tendency (e.g. means) or other basic estimates (e.g. regression coefficient) AND variation (e.g. standard deviation) or associated estimates of uncertainty (e.g. confidence intervals)
☐	☒	For null hypothesis testing, the test statistic (e.g. F, t, r) with confidence intervals, effect sizes, degrees of freedom and P value noted<br>*Give P values as exact values whenever suitable.*
☐	☒	For Bayesian analysis, information on the choice of priors and Markov chain Monte Carlo settings
☒	☐	For hierarchical and complex designs, identification of the appropriate level for tests and full reporting of outcomes
☐	☒	Estimates of effect sizes (e.g. Cohen's d, Pearson's r), indicating how they were calculated

Our web collection on statistics for biologists contains articles on many of the points above.

Software and code

Policy information about availability of computer code

Data collection	No software was used for data collection.
Data analysis	MEFISTO is implemented as part of the MOFA framework, which is available as Bioconductor package MOFA2 and at https://github.com/bioFAM/MOFA2. Code to reproduce all figures is available at https://github.com/bioFAM/MEFISTO_analyses. In addition, we provide vignettes on the main applications as part of the MEFISTO tutorials on https://biofam.github.io/MOFA2/MEFISTO.<br><br>For data analysis the following Python (python=3.8.8) packages were used: argparse==1.4.0, cycler==0.10.0, dtw-python==1.1.6, gpytorch==1.4.0, h5py==3.1.0, joblib==1.0.1, kiwisolver==1.3.1, matplotlib==3.3.4, numpy==1.20.1, pandas==1.2.3, pillow==8.1.1, pyparsing==2.4.7, python-dateutil==2.8.1, pytz==2021.1, scikit-learn==0.24.1, scipy==1.6.1, seaborn==0.11.1, six==1.15.0, threadpoolctl==2.1.0, torch==1.7.1+cpu, torchaudio==0.7.2, torchvision==0.8.2+cpu, typing-extensions==3.7.4.3, gemelli==0.0.5<br><br>The following R (R 4.0.0 and R 4.1.0) packages were used: motifmatchr_1.12, scran_1.18, magrittr_2.0.1, cowplot_1.0.1, forcats_0.5.0, stringr_1.4.0, dplyr_1.0.0, purrr_0.3.4, readr_1.3.1, reshape2_1.4.4, tidyr_1.1.0, tibble_3.0.2, ggplot2_3.3.2, tidyverse_1.3.0, BiocStyle_2.16.0, SeuratObject_4.0.0, Seurat_4.0.0, lmerTest_3.1.3, SeuratData_0.2.1, Seurat_3.2.3, MOFAdata_1.6.0, ggrepel_0.9.1, ggpubr_0.4.0, data.table_1.13.6, DESeq2_1.26.0<br><br>For microbiome analysis additionally qiime2-2020.8 and iTOL v6 was used.

For manuscripts utilizing custom algorithms or software that are central to the research but not yet described in published literature, software must be made available to editors and reviewers. We strongly encourage code deposition in a community repository (e.g. GitHub). See the Nature Research guidelines for submitting code & software for further information.

Data

Policy information about availability of data

All manuscripts must include a data availability statement. This statement should provide the following information, where applicable:

- Accession codes, unique identifiers, or web links for publicly available datasets
- A list of figures that have associated raw data
- A description of any restrictions on data availability

The evodevo data was obtained from Cardoso-Moreira et al (10) and can be accessed from ArrayExpress with codes E-MTAB-6782 (rabbit), E-MTAB-6798 (mouse), E-MTAB-6811 (rat), E-MTAB-6814 (human) and E-MTAB-6833 (opossum) (https://www.ebi.ac.uk/arrayexpress/). The microbiome data is based on Bokulich et al (25) and can be found on Qiita (http://qiita.microbio.me), the processed data was obtained from the 'Code Ocean' capsule: https://doi.org/10.24433/CO.5938114.v1 provided by Martino et al (26). The scNMT-seq data was obtained from Argelaguet et al (29) and the spatial transcriptomics data set from the SeuratData package under the name stxBrain.anterior1.

Processed data and trained models for all applications are available at https://doi.org/10.6084/m9.figshare.13233860.v1 as used in the tutorials at https://biofam.github.io/MOFA2/MEFISTO.

Enrichment analyses were based on gene and marker sets available from the Bioconductor package MOFAdata v1.6.0 (including MSigDB (33) and Reactome (53) gene sets) and from PanglaoDB (https://panglaodb.se/), TF motifs were extracted from the JASPAR database (57).

Field-specific reporting

Please select the one below that is the best fit for your research. If you are not sure, read the appropriate sections before making your selection.

☒ Life sciences ☐ Behavioural & social sciences ☐ Ecological, evolutionary & environmental sciences

For a reference copy of the document with all sections, see nature.com/documents/nr-reporting-summary-flat.pdf

Life sciences study design

All studies must disclose on these points even when the disclosure is negative.

Sample size	No pre-determination of sample size was required, as no data was generated for this study and no hypothesis-based experiment performed. Sample size was used as available from original studies and set in simulations to numbers that reflect dimensions seen in existing data sets.
Data exclusions	No data was generated for this study. During data preprocessing filters were applied as detailed in Methods section.
Replication	No data was generated for this study. For computational analysis, we provide all code as open-source ressource to ensure reproducibility and, where relevant, multiple random seeds where used for computational analyses and performance assessment.
Randomization	No sample randomization was required, as no data was generated for this study. For computational analyses and method evaluations, samples and features were randomly selected, generated or masked as detailed in Methods.
Blinding	No data was generated for this study and blinding is not applicable, as this study illustrates different applications and does not test a specific hypothesis.

Reporting for specific materials, systems and methods

We require information from authors about some types of materials, experimental systems and methods used in many studies. Here, indicate whether each material, system or method listed is relevant to your study. If you are not sure if a list item applies to your research, read the appropriate section before selecting a response.

Materials & experimental systems

n/a	Involved in the study	
☒	☐	Antibodies
☒	☐	Eukaryotic cell lines
☒	☐	Palaeontology and archaeology
☒	☐	Animals and other organisms
☒	☐	Human research participants
☒	☐	Clinical data
☒	☐	Dual use research of concern

Methods

n/a	Involved in the study	
☒	☐	ChIP-seq
☒	☐	Flow cytometry
☒	☐	MRI-based neuroimaging
